# Homogenized and stigmatized: A discourse analysis of Asian sub-ethnic medical school aspirants

**DOI:** 10.1371/journal.pone.0335860

**Published:** 2025-10-31

**Authors:** Salman H. Choudhry, Keegan D’Mello, George Kim, Robin Mackin, Amrit Kirpalani

**Affiliations:** 1 Schulich School of Medicine & Dentistry, Western University, London, Ontario, Canada; 2 Lawson Health Research Institute, London, Ontario, Canada; Xi'an University of Posts and Telecommunications, CHINA

## Abstract

The study examines the influence of Asian sub-ethnic identity on the experiences of pre-medical students in the United States and Canada, aiming to understand how early interactions with the medical education system shape their pursuit of medicine. The researchers analyzed 132 discussion threads from popular online premedical school forums between June 2018 and 2023. The Asian Critical Theory framework guided the analysis along with cyclical inductive coding. Two major themes emerged: the homogenization of diverse Asian sub-ethnicities and external pressure related to sociocultural values. Terms like “over-represented minorities” contributed to the perception of Asians as a monolithic group, while expressions such as “Asian Parents” highlighted unique familial expectations. Non-Asian users often dismissed these barriers, reinforcing the model minority myth. The study emphasizes the negative consequences of framing Asians as a homogenous group in medical school admissions policies, perpetuating stereotypes, and overlooking the diversity within Asian sub-ethnic communities. The term “overrepresented” is critiqued for its role in homogenizing Asian identities and undermining the complexity of their experiences. These findings highlight the need for greater recognition of the nuanced challenges faced by Asian sub-ethnic medical trainees and the importance of dismantling stereotypes in medical education.

## Introduction

Efforts to promote equity in medical education often overlook the distinct challenges faced by Asian sub-ethnic students in North America. These students are frequently subject to the “model minority” myth, a stereotype that portrays Asians as inherently intelligent, hardworking, and academically successful [1]. This perception reinforces a racialized narrative of biological advantage, contributing to the homogenization of diverse Asian identities and the categorization of many Asian applicants as “overrepresented” in higher education [2–4]. As a result, Asian students are often excluded from diversity initiatives and may encounter unique barriers long before entering medical school [3].

In clinical settings, Asian medical students and residents report being ostracized, stereotyped, or given fewer learning opportunities due to their racialized identity [5–7]. While such marginalization is well-documented at advanced stages of training, less is known about how Asian sub-ethnic identity influences the earlier stages of professional development. Premedical students may internalize or resist racialized expectations, often expressed in candid ways on anonymous online forums.

One label commonly used in these forums is “ORM” (Overrepresented Minority), a non-official designation reflecting the perception that Asian applicants are held to higher admissions standards. These online discussions provide a window into how structural inequities in medical education are understood, reinforced, or challenged by premedical students.

The premed community provides an early time point at which students have decided (or are strongly considering) to pursue medical education, with many beginning to show signs of professional identity formation [8]. Previous work has shown that online forums are an invaluable tool for exploring the discourse around the intersection of racialized identity and medical school admissions in an anonymous setting [9]. These thread-based websites and forums provide open communication between users on career advising, tools, peer support, and criticism related to the medical school journey.

This study explores how Asian sub-ethnic identity influences the discourse of North American premedical students navigating medical school admissions. Through discourse analysis of online forum posts, we aim to better understand the racialized assumptions embedded in medical education and highlight the experiences of Asian premedical communities.

## Methods

We conducted a two-stage qualitative approach combining content analysis and critical discourse analysis (CDA) to examine online discussions about Asian identity among North American premedical students. Posts were drawn from four major anonymous forums: Reddit (r/premed and r/premedcanada), Student Doctor Network (SDN), and Premed101. These platforms are widely used for peer advice and candid discussion of medical admissions. Our approach aimed to identify recurring themes and analyze how language shaped and reflected racialized assumptions within the premedical landscape.

Content analysis served as the initial phase of our methodology, allowing us to collect and categorize textual data from these forums. Content analysis was informed by the principles laid out by Hardy et al (2004) that acknowledge the potential for nuanced meanings and contexts within the text.

CDA was then used to interpret the discussion texts and categorized data. CDA acknowledges that social habits are inseparable from language, so the implicit social and power dynamics in discourse can reveal what is considered acceptable in social situations [10].

### Methodologic coherence

We acknowledge the distinct epistemological assumptions underlying content analysis and CDA. Content analysis assumes a relatively stable and consistent meaning of words and phrases, focusing on systematic categorization [11]. In contrast, CDA views language as dynamic and context-dependent, emphasizing the power relations, sociocultural ideologies, and implicit practices embedded in discourse [10]. These differing assumptions about data, meaning-making, and interpretation presented potential methodological tensions, which we addressed through deliberate integration and alignment with the study’s research objectives.

To reconcile these differences, we employed a two-stage approach designed to capitalize on the complementary strengths of these methods [11]. Content analysis was selected to systematically identify recurring patterns and domains, offering a structured foundation for understanding the linguistic context of specific terms. This method aligned with the research objective of uncovering dominant themes within premedical discourse. CDA then facilitated a deeper exploration of how these patterns reflected or perpetuated systemic biases, power dynamics, and sociocultural ideologies. This second phase aligned with the objective of examining the underlying societal and structural forces influencing discourse. This integrated approach allowed us to comprehensively address both the prevalence and the significance of key themes in the data.

To ensure methodological coherence, we grounded both methods in the Asian Critical Theory (AsianCrit), which provided a lens through which to interpret themes identified in content analysis and further contextualize them in discourse analysis. This alignment minimized potential tensions between methods by situating both within a shared theoretical foundation.

We also employed iterative triangulation and reflexivity (as described below) throughout the research process to align the methods and address any tensions. This iterative process ensured that content analysis and CDA complemented one another in addressing the study’s research questions, providing a coherent and integrated analytical framework.

### Theoretical framework

The guiding theoretical framework for this study was AsianCrit, which addresses the experiences and voices of Asian Americans and challenges the white master narrative [12]. AsianCrit is grounded in the experiences and voices of Asian Americans, and centers on the racial realities of Asians in the Global North. The AsianCrit framework examines Asian sub-ethnic communities issues in education through seven key tenets: 1) Asianization, the racialization process of Asians in the Americas as “perpetual foreigners’‘, 2) Transnational Contexts, historical legacies shaped by economic, political, and social processes that extend beyond national borders, 3) (Re)constructive History, re-analyzing history from the perspective of Asian Americans, offering a counter-narrative to the dominant voice, 4) Strategic (Anti)essentialism, use of identity labels such as Asian American as a tool of sociopolitical agency and resistance, while recognizing the diversity and complexities within that label, 5) Intersectionality, considering how layers of marginalized social identities (e.g., gender, class, ability status) inform experiences, 6) Story, Theory, and Praxis, the importance of stories and counter-stories of Asian sub-ethnic communities, 7) Commitment to Social Justice, advocating for the rights and equality of Asian sub-ethnic communities in the Global North consisting of the United States and Canada [12]. These tenets allow for a nuanced understanding of how white supremacy shapes the lives of Asian sub-ethnic individuals in the Western world.

The AsianCrit framework guided the research design by focusing on how racialization, intersectionality, and resistance shape the experiences of Asian sub-ethnic premedical students. In data collection, the framework led us to select anonymous online forums to capture unfiltered discourse that included both dominant stereotypes and critical counter-narratives. In content analysis, it directed our focus to recurring linguistic signs of marginalization or identity erasure. In discourse analysis, it allowed us to explore how dominant narratives are reproduced or resisted through online speech and helped identify recurring themes such as Asianization and the model minority myth and emphasized the importance of amplifying marginalized voices. AsianCrit was particularly valuable in recognizing the sociocultural specificity of sub-ethnic Asian identities, and how these identities are often flattened into a monolithic “Asian” category in medical admissions discourse.

### Data collection

**Fig 1:**
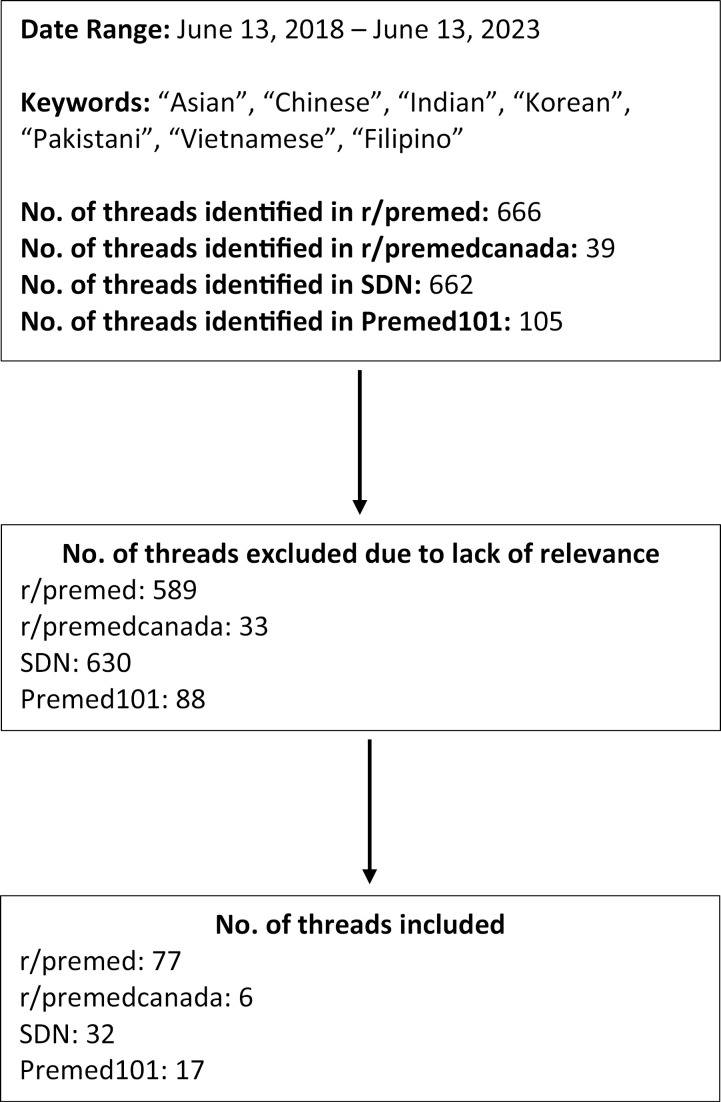
Flowchart of thread selection.

Original postings and responses relevant to Asian sub-ethnic identity on online forums of r/premed, r/premedcanada, SDN, and Premed101 were examined. These forums are organized into threads which included an anonymous user posting an initial comment or question, and further discussions, questions, and comments are included by other anonymous online forum users. Initially, an author (SHC) completed a preliminary scan of discussion forum threads from June 13, 2018 to June 13, 2023 to identify the general online discourse regarding Asian sub-ethnic identity. Based on our review of these threads and the existing literature, a set of keywords related to Asian identity was then constructed based on relevance to the primary research aim (Fig 1). Using predefined keywords, we identified relevant threads from four online forums between June 2018 and June 2023. We selected threads focused on Asian sub-ethnic identity in the context of medical admissions for further coding. We excluded threads that did not directly address issues related to Asian sub-ethnic identity or lacked discourse on medical school admissions. For example, threads focusing solely on general academic advice without racial context, personal anecdotes unrelated to race, or discussions around non-Asian ethnic groups were excluded.

We conducted a two-stage qualitative analysis using content analysis and inductive coding. Content analysis categorized textual data from online forums, identifying recurring themes and domains (see Table 1). This stage provided an overview of the frequency and context of relevant terms, helping to map common themes and linguistic patterns. Content analysis helped establish the major categories and domains within the data, which served as the foundation for deeper analysis. Following content analysis, the inductive coding process was done by two authors (SHC and KD), allowing for a more nuanced, flexible examination of the data. Researchers independently coded forum posts, and the preliminary codes were compared between the two researchers. After confirming similarity in coding patterns, both team members coded the remaining threads. The coding process was cyclical in nature as researchers returned to the data following an initial coding pass to refine and group codes to highlight similarities and minimize redundancy. This iterative process helped identify underlying themes, power dynamics, and social contexts not captured by the initial content analysis.

**Table 1:** Categories and domains derived from content analysis.

Category	Keywords	Description	Domain
**Homogenization of Asian Identity**	“ORM” (Overrepresented Minority), “Asian,” “Pan-ethnic,” “Asianization”	Discussions around the amalgamation of diverse Asian sub-ethnicities into a single racial category.	Homogenizing of Asians
**Model Minority Myth**	“Model Minority,” “Academic Performance,” “Higher Standards,” “Overachievement”	Stereotypes about Asians as academically superior, leading to perceptions of higher admission standards.	Stereotypes and Biases
**Parental Pressure**	“Asian Parents,” “Tiger Parents,” “Strict Parenting,” “Academic Success”	Discourse related to sociocultural and familial expectations influencing premedical students’ paths.	Asian Sub-ethnic Values on Physicianship
**Overrepresentation in Medicine**	“URM” (Underrepresented Minority), “Viability for Applications,” “Discrimination”	Confusion regarding whether certain Asian sub-ethnicities are URM or ORM in medical school admissions.	Racial Categorization and Representation
**Intersectionality and Identity**	“Gender Roles,” “Family Expectations,” “Career vs. Family,” “Immigrant Experience”	Discussion of challenges faced by individuals with multiple marginalized identities within Asian sub-ethnic groups.	Intersectional Challenges in Medical Training

A group content analysis was initially performed by three researchers (SHC, KD, AK) during a group discussion and meeting. We then employed triangulation to involve two additional researchers (GK, RM) further removed from the initial data collection to provide further perspective and refinement of the content analysis. Within each domain collated from the content analysis, we then conducted our critical discourse analysis to explore the impact of language on the social context.

### Critical discourse analysis

CDA served as the second stage of our two-part analytic approach. CDA is a qualitative methodology that views language as a social practice and assumes that discourse both reflects and shapes power structures, ideologies, and social norms. CDA emphasized the contextual meaning of language, focusing on how it constructs, legitimizes, or challenges social inequities.

In this study, CDA allowed us to examine how discourse on online forums refinforced or contested the model minority myth, the ORM label, and other racialized beliefs about Asian sub-ethnic applicants. Drawing on foundational work by Strauss and Feiz (2014), we interpreted posts not only for their surface meaning but also for their role in maintaining or resisting dominant power relations. CDA was well suited to analyzing anonymous discourse where users often expressed views that may be supresed in public or institutional settings.

The discourse analysis was an iterative process, informed by the preliminary findings from our content analysis (see Table 1 for identified categories and domains). After identifying key themes through content analysis, we transitioned into a more detailed examination of how language used in the forums reinforced or challenged power dynamics. In this phase, we specifically examined how users’ language reflected societal beliefs about Asian identity in medical school admissions, looking for implicit social practices that perpetuated racial biases and stereotypes.

Two authors (SHC and KD) initially conducted the discourse analysis by coding posts for instances of racialization, marginalization, and resistance. A collaborative approach was employed during team discussions to refine the themes, with additional input from GK, RM, and AK, who provided critical perspectives removed from the initial coding phase. This triangulation helped enhance the reliability of the findings and ensured that multiple viewpoints were considered. To further improve the credibility of the results, we practiced reflexivity.

### Reflexivity

The authors in this study originate from different ethnic backgrounds, with each author having a different Asian sub-ethnicity. The authors also have diverse educational backgrounds: faculty physicians, medical students, and education researchers. All authors share a common interest in recognizing how Asian identity influences the medical education journey. The authors reflected individually and as a group on their own backgrounds, biases, and the potential impacts of these biases on the data analysis. We regularly identified and challenged our own assumptions and beliefs to mitigate the impact of our own biases, though recognize that our biases may impact our interaction with the data.

### Ethics

In accordance with Western University protocol and Tri-Council Policy Statement (TCPS) Article 2.2, this qualitative study was exempt from ethics board approval since no direct contact was made with users and it did not pose any risk to or adversely affect the welfare of any individuals. All data were collected from publicly accessible online forums in compliance with their respective terms of service. No usernames or identifiable data were collected. No interventions or interactions with users occurred. Forum content was analyzed in accordance with TCPS Article 2.2.

## Results

A total of 132 discussion threads from r/premed (n = 77), r/premedcanada (n = 6), SDN (n = 32), and Premed101 (n = 17) pertaining to Asian identity in the premedical population were included in the analysis (Fig 1) with a total of 447 individual posts. While most users remained anonymous, some identified their racialized status, identified whether they were current medical students or aspirants, and stated their level of training as undergraduate or graduate students. Content analysis of the online threads revealed two major domains of discussion that provide insight into how Asian sub-ethnic identity influences the experiences of premeds pursuing medical education in Canada and the United States. The first was the homogenization of Asians by medical institutions. This resulted in confusion regarding identity, discussion around status as underrepresented in medicine (URM) or overrepresented in medicine (ORM), and prominent biases and stereotypes against Asians. The second major domain revolved around the influence of Asian sub-ethnic values on physicianship, with Asian-identifying students expressing a great deal of external pressure, mainly from their parents, families, and socio-cultural expectations.

### Domain 1: Homogenizing of Asians

The most prominent discussion across all forums centered around the perceived amalgamation by the medical education community of all Asian sub-ethnicities into one all-encompassing group. Homogenization, reflecting the AsianCrit concept of Asianization, occurred when applicants had to identify only as ‘Asian,’ without sub-ethnic options. Self-identifying Asian sub-ethnic users felt that medical school applications may have inaccurately or incompletely identified them to admissions committees. This pan-ethnic grouping also caused a significant amount of confusion amongst forum users in determining their viability for medical school applications and was also highlighted by users as a leading cause for them having been victimized by stereotypes.

#### Perceived overrepresentation and the ORM label.

The institutional homogenization of Asian sub-ethnicities sparked discussion about which groups are classified as URM or ORM. Through the AsianCrit lens, these conversations reflected the perception that the diversity within Asian communities was being erased in favor of a pan-ethnic label. From a thread entitled “Does the “type” of Asian ORM Make a difference” many users concurred with the belief that *“... they are all lumped together and treated as OR(M).*” Specific terms such as “ORM” were often used when individuals were seeking advice for their application and chances of matriculation into medical school or often by others to dismiss the concept of institutionalized racism. The label ‘ORM’ reflected the perceived Asianization of these individuals, with many feeling marginalized and overlooked by admissions committees.

*“I hate the designation ORM with a burning passion. It’s basically a “sorry we don’t need any more of you Yellows in med school” but everyone treats it as ok”*

*“I have good scores and good extracurriculars, but … I am also really nervous that because I am Indian, med schools will just look at my application like another stereotypical ORM app.”*

Conversely, some users went on to state that Asian-identifying ORMs “victimize” themselves and that there is no systemic discrimination in place. This was emphasized by discourse minmizing the effects of institutional homogenization.

*“You missed the part where Asians are over represented by a factor of three times higher than they are in the population. I fail to see any argument where this is institutionalized racism.”*

*“ORM applicants usually have better resources and support from family, school in their neighborhoods, and society in general”*

Institutional homogenization of Asian sub-ethnicities led to confusion about whether certain groups were classified as URM or ORM. This was a substantial cause for confusion and conversations pointed out how certain Asian sub-ethnicities such as Thai, Vietnamese, Cambodian, and many more are technically URM. Individuals who identified as Asian but were URM discussed their own academic journeys and emphasized how their success within their own sociopolitical context was often minimized due to their ethnicity and URM status: *“Wait till people downplay your accomplishments and attribute your spot in med school to being URM…”.* This rhetoric once again intersects with the notion that transnational contexts were perceived by some as unimportant in the admissions process.

#### Stereotypes and higher standards in admissions.

Forum users who identified with an Asian sub-ethnicity reported facing a higher standard to succeed in applying to medical school than other applicants. *“Unfortunately, it is the ORM/ Asian American meritorious students who have to prove their “mission” and “motivation”*. The most significant discourse included Asian-identifying students requiring “higher stats” (GPA and MCAT scores) to be considered competitive and a general consensus that the medical school application process is more difficult for Asian-identifying students. When Asian-identifying applicants sought advice or feedback on their application, the discourse centered around the need to meet “higher”, “better” or, “above average [compared to non-Asian]” standards.

*“Based on what I’ve read so far… the standard is a lot higher for Asians (I believe it’s called ORM applicants) to get accepted”*

This language was tied to the perpetuated belief that Asian applicants as a pan-ethnic group are perceived as being academically superior amongst the applicant cohort, and thus users suggested that the relative selection criteria would be different amongst “ORMs.” Comments from self-identified Asian users revolved around the tenet of anti-essentialism, wherein users suggested that distancing oneself from the pan-ethnic Asian groups would be beneficial given the ingrained nature of the model minority myth within the medical school admissions process:

*“...schools select so hard against Asian applicants it’s like old school racism.*

*“So, Asians are, on average, higher performing than non-Asians. So, on Average, [Admissions Committees] (ADCOMs) are exposed to more high performing Asians, so they develop a schema unintentionally via exposure. This leads to an implicit bias.”*

These comments supported the notion of foundational racism within the medical education (and general higher education) system.

#### Dismissal and resistance in online discourse.

When users expressed their discontentment with this systemic racial inequity, it was often met with backlash by anonymous users who stated that attributing lack of acceptance into medical school due to race was a victim mindset. Comments suggested self-identified Asian applicants should “stop worrying about it” or else stop “being sensitive”, suggesting resistance within the premedical communities to acknowledging barriers facing these students.

Discourse also revolved around prevalent negative stereotypes associated with Asians, including the generalization that all Asians want to be doctors. Users pointed out how the homogenization of Asians by medical education and the resultant “ORM” classification was cyclical with the stereotype that “Asians want to be doctors” and the tougher admission process faced by Asian students.

*“...Thus, the repeated and continued internalization by Asians that they must perform better because of their race fuels the perpetuation of the biases in an ever stronger and cyclic manner…”*

*“...For these Asian students, their “average” performance (performance on par with other racial groups) will end up hurting them by no fault of their own. Pointing to other social and cultural factors here such as higher work ethic, higher value placed on education, etc to justify these admissions practices would be shortsighted…”*

These conversations and the language used reinforce the perceived Asianization while also providing counter-narratives from the Asian sub-ethnic communities, suggesting that systemic racism is the main driver of these perpetuated beliefs. These voices against dominant narratives shed light on the social context in which Asian-identifying premedical students viewed their identities, suggesting stereotypes are deeply ingrained within medical education and the broader premedical community.

### Domain 2: Asian sub-ethnic values around physicianship

#### Parental and familial expectations.

Asian sub-ethnic users frequently discussed parental pressure, often emphasizing success in academics at the cost of personal well-being. This was emphasized with the mention of parents taking “control” or else forcing their children down an academic pathway, highlighting a lack of autonomy and independence from their.

“I can’t do this s*** I’m tired of being controlled by my parents…”

*“My Asian parents pushed me to become a doctor all the time growing up and I would tell them no over and over again…”*

*“My asian parents keep questioning me every day about how many secondaries I’ve submitted… It seems like my parents want me to get into med school more than I want to get into med school”*

A strict parenting style was denoted as “tiger parents”; a stereotype associated with parents heavily invested in their child’s academic achievements, which pressured students to achieve greater academic credentials. This discourse again highlighted the need of reconstructive history, with users offering the oft-overlooked perspective from the lens of Asian sub-ethnic communities in the Global North. Forum users identified how the societal portrayal of Asian students as diligent academic achievers in medical education contributed to increasingly stringent expectations by both family and medical institutions.

Discourse also revealed students experienced a lack of encouragement and appreciation from parental figures. Forum users pointed out how Asian parents are “momentarily satisfied” with academic accomplishments before demanding greater expectations: *“Haha, jokes on you… they can’t wait to pick a specialty for you….”* This language highlights the cyclical and pervasive nature of sociocultural influences on medical school aspirations, which appeared to be a recurring theme in the counter-narratives offered by Asian sub-ethnic students.

#### Cultural norms and professional aspirations.

Beyond parental pressure and expectations, there was significant discourse around experienced pressure from extended families and cultural norms. Posters noted that both immediate and extended families often criticized students in their choice of school, academic performance, or the selected program. Users discussed the challenge of establishing boundaries with Asian family members, emphasizing the difficulty of imposing such boundaries in Asian culture. Discourse spotlighted the idealization of physicians (“Doctors are like Gods in India”) in Asian culture, further perpetuating the stereotype that all Asians aspire to be doctors.

#### Gender roles and intersectional pressures.

Whereas comments on this topic ranged from emotional discussion to humor-filled posts (i.e., jokes or memes about “Asian parents”), the discourse further expanded upon the prominent counter-narrative (reconstructive history) from the Asian voices, emphasizing the difficult process experienced by premedical students because of cultural expectations and criticisms imposed by family. Some users specifically commented on the underrecognized challenges from intersectional identities, such as those related to perceived gender roles within their cultural context:

*“Ever since getting into medical school the comments and questions I get are: How will you balance your career with your family? Don’t choose xyz specialty because you won’t have time for your kids. When will you get married and have kids if you’re always in school and or training? I come from a traditional south Asian family and hear these comments all the time. I’m the first woman in my family to actually pursue a career. I keep getting these questions and it really drains me thinking about the future and the fact that I have to choose one or the other.”*

In response to these posts, several self-identified white users suggested that the familial expectations of Asian communities were the underlying cause for the hardships facing these students during the application process. From a thread titled, *“Getting a 4.0 isn’t good enough for my Asian Parents”* one user wrote *“Dude sorry but I hate Asian parents. They’re the reason why Asians have it so hard getting into med school, and why the average matriculant GPA & MCAT is so inflated.”*

Discourse in this area underscored the socialization process that results from Asianization. With hardships being directed towards Asian parents and families rather than systemic racism, the discussions suggested an internalization of the model minority myth which may fuel sociocultural expectations within Asian sub-ethnic communities, while also providing an opportunity for those in privileged positions to dismiss barriers facing these populations.

## Discussion

Our analysis of discourse sheds light on the multifaceted challenges faced by premedical students of Asian sub-ethnic backgrounds as they navigate the complex terrain of medical education in Western-centric environments. Drawing upon the Asian Critical Theory (AsianCrit) framework, we found prominent examples of systemic racism and anti-Asian stereotypes deeply ingrained within medical education and the broader premedical community.

Our findings highlight the harm of framing ‘Asians’ as a homogenous group in admissions, with ‘ORM’ driving Asianization. Though the term appears to have been normalized in the premedical community, scholars have long contended that the Asianization process contributes to the dehumanizing of Asians in North America [13], suggesting that such language may reinforce racist infrastructures stemming from white supremacy [14]. Whereas the term “URM” has been revised due to inadequate recognition of diverse identities [15], our results suggest that “ORM” itself is a term that deserves reassessment and may require proactive dismantling within the premedical community, particularly given its use to perpetuate the monolithic identity of Asian sub-ethnicities and ignore multiple sub-ethnic identities. Moreover, as it has previously been identified that medical schools’ messaging around diversity in admissions often fails to capture the true importance and societal value of diversity and inclusion [16], the present study suggests that the concept of Asians as “ORMs” ignores transnational contexts within these communities, further diminishing the diversity of the applicant cohort itself. Being perceived as a monolith, Asian-identifying premedical students confront a distressing conundrum, questioning whether their academic accomplishments and medical school applications can transcend the label of being “ORM”.

The concept of “Asians” as a panethnicity also reinforced stereotypes about these sub-populations which appeared to trigger a sense of ‘otherness.’ Given that experiences of otherness and ostracism are profound for Asian sub-ethnic clinical trainees [1], our findings suggest that the foundation for these experiences may be developed even before medical school. In line with previous literature on senior medical trainees [5], the present study supports that a substantial driver of these discriminatory beliefs and homogenization is the model minority myth - both ingrained in higher education, but also reinforced by students’ own familial sociocultural experiences. To combat this phenomenon, medical schools should consider disaggregating Asian sub-ethnic populations in their data collection and public-facing material. In addition, they can implement several strategies such as tailored support programs for Asian sub-ethnic applicants, cultural competency training for admission committees to ensure a more equitable evaluation process, and inclusive admission policies. Moreover, institutional leaders should be aware of the harmful nature of the “ORM” label and consider discouraging use of this term in discourse around admissions. While our findings focus on premedical students in North America, they have broader implications across various regions and medical schools. The challenges of homogenization, cultural pressures, and systemic bias are not unique to North America and likely affect Asian sub-ethnic groups in other contexts. Schools across different countries can apply our recommendations to diversify admissions policies**,** particularly in regions where Asian applicants face similar stereotyping and exclusion.

Our study also found that Asian sub-ethnic students offer their own reconstructed history within the framework of AsianCrit – providing the voice of their own lived experiences to run counter to dominant narratives. Whereas these stories were used to provide critical counterarguments to anti-Asian sentiment, we found that they were often met with reinforced beliefs in the model minority myth, with non-Asian users suggesting Asian communities were academically unparalleled. In line with findings across higher education research, students employed the model minority myth as a strategic tool to dismiss barriers facing Asian sub-ethnic communities by strategically positioning them as intrinsically advantaged [17]. In many instances, we note that self-identified Asian students themselves reinforced these beliefs, which is of additional concern as internalization of the model minority myth has been linked to psychological distress within Asian sub-ethnic communities [18]. Given the detrimental impacts of these stereotypes on well-being amongst physicians in training [5], a deeper, focused exploration of the model minority myth within the context of medical education is warranted.

Strengths of this study include the use of both content and discourse analyses to provide a more nuanced understanding of the discussion. Moreover, the anonymous nature of all forums allowed us to reduce the risk of social desirability or response bias in the analyzed discourse. We do note key limitations including a predominance of United States-based discourse as the amount of content on American forums was much larger than Canadian content. We also note that while our search allowed us to capture a wide breadth of discourse around Asian identity, the Asianization phenomenon inherently reduces our ability to further explore the identities and experiences of all diverse communities. Moreover, we acknowledge that within the many Asian sub-ethnic communities there are also unique phenomena not captured in the present study that would deserve their own focused exploration such as Islamophobia, immigration, language barriers, and an expanded view of intersectionality.

Our findings extend existing literature by offering unique insights into how the homogenization of Asian sub-ethnic identities in medical admissions perpetuates systemic biases. Unlike prior studies, we demonstrate how terms like “ORM” obscure the diversity within Asian communities, which has significant implications for policy reform in admissions. Additionally, we highlight the impact of Asian sub-ethnic values on premedical students’ career paths, showing how familial and cultural pressures uniquely shape their experiences, a perspective often underexplored. By applying AsianCrit, we also underscore the need for disaggregating Asian identities in both research and institutional practices, advancing the discourse on intersectionality and systemic racism in medical education.

In conclusion, this study reveals the complex challenges faced by Asian sub-ethnic medical students in admissions. By employing the AsianCrit framework, we highlight deeply ingrained anti-Asian stereotypes. The findings underscore the urgent need for a more nuanced disaggregation of Asian identities within medical education and a reassessment of the term “ORM” within the premedical community.
